# Comparison of Kaltenborn-Evjenth, McKenzie, and HVLA Manipulation Techniques in the Treatment of Lumbar Spine Pain: A Review of the Literature

**DOI:** 10.3390/healthcare13192403

**Published:** 2025-09-24

**Authors:** Michał Grzegorczyk, Magdalena Brodowicz-Król, Grażyna Brzuszkiewicz-Kuźmicka

**Affiliations:** 1 Department of Rehabilitation and Physiotherapy, Faculty of Health Sciences, Medical University of Lublin, 20-081 Lublin, Poland; 2 Faculty of Health Sciences and Psychology, Collegium Medicum, University of Rzeszów, 35-959 Rzeszow, Poland; mbrodowicz@ur.edu.pl; 3 Department of Clinical Physiotherapy, Faculty of Rehabilitation, Józef Piłsudski University of Physical Education in Warsaw, 00-968 Warsaw, Poland; grazyna.kuzmicka@awf.edu.pl

**Keywords:** lumbar back pain (LBP), physiotherapy, McKenzie method, HVLA manipulation, Kaltenborn-Evjenth method, effectiveness of special physiotherapy methods

## Abstract

Lumbar spine pain (LBP) is a leading cause of disability worldwide and remains a major challenge in clinical practice. Among non-invasive treatment strategies, manual therapy plays a central role, offering individualized interventions that target both biomechanical dysfunction and pain. This narrative review compares three commonly used physiotherapeutic approaches—Kaltenborn-Evjenth mobilization, the McKenzie method, and high-velocity low-amplitude (HVLA) manipulation—based on current evidence regarding their effectiveness, safety, and clinical application. A total of 32 randomized controlled trials, systematic reviews, and meta-analyses published between 2003 and 2024 were analyzed. The Kaltenborn-Evjenth method demonstrated notable effectiveness in improving range of motion and reducing chronic pain, particularly in patients with segmental hypomobility. The McKenzie method showed strong outcomes in both acute and chronic LBP, especially in cases involving symptom centralization and high patient engagement. HVLA techniques offered rapid symptom relief in acute phases but required careful patient selection due to their mechanical intensity. The findings suggest that no single method is universally superior. Instead, optimal outcomes are achieved through individualized treatment plans that integrate multiple techniques based on clinical presentation, pain chronicity, and functional limitations. Multimodal strategies that combine manual therapy with exercise and patient education appear to be the most effective in managing LBP and preventing recurrence.

## 1. Introduction

Low back pain (LBP) is one of the most common musculoskeletal disorders, leading to disability, absenteeism from work, and decreased quality of life [1]. The complex pathogenesis, ranging from biomechanical overload to postural and psychosocial disorders, means that treatment of lumbar back pain requires individualization and methods that act both symptomatically and causally. Epidemiological data indicate that up to 70–80% of adults experience an episode of low back pain at least once in their lifetime. In many cases, the symptoms resolve spontaneously; however, in some patients, they become chronic, leading to long-term functional limitations, work absenteeism, and a reduced quality of life.

The development of the special methods department of physiotherapy stemmed from the need to treat pain non-invasively, on an individual basis, and taking into account functional causes. Manual therapy, including manipulation, mobilizations, musculoskeletal techniques, trigger point therapy, and concepts such as the McKenzie method, is now an important element in the therapeutic treatment of physiotherapists, osteopaths, and manual medicine physicians. With the increasing number of patients with lumbar spine pain syndrome and the limited effectiveness of pharmacological therapy, special methods of physiotherapy are playing an increasingly important role. Among the most commonly used are: (1) Mobilizations using the Kaltenborn-Evjenth method, (2) McKenzie method (MDT—Mechanical Diagnosis and Therapy), (3) HVLA (High-Velocity Low Amplitude) manipulations.

Each of these techniques is based on different therapeutic principles and is suited to distinct clinical presentations. The Kaltenborn-Evjenth method is primarily indicated for patients with segmental hypomobility, joint stiffness, and chronic mechanical pain that is resistant to general exercise. It is often used when low-velocity, graded mobilizations are needed to restore joint play and reduce soft tissue tension without triggering pain. The McKenzie method is most appropriate for individuals with mechanical derangement syndromes, particularly when symptom centralization or directional preference is observed. It is well-suited for patients who can participate in active therapy and benefit from self-management strategies. HVLA manipulation is typically applied in acute or subacute cases with restricted spinal mobility and no contraindications, offering fast relief through neurophysiological stimulation and segmental release.

Accurate diagnosis and appropriate patient selection are crucial to the success of these interventions. Differential diagnosis of lumbar spine conditions must consider not only mechanical causes but also potential red flags such as infections, fractures, tumors, or inflammatory disorders. Clinical screening tools, including centralization signs, motion restriction tests, neurological examination, and psychosocial risk assessments, help determine the underlying mechanism of pain and guide the choice of therapy. Misclassification can lead to suboptimal outcomes or adverse effects, particularly in high-velocity techniques. Therefore, individualized assessment remains the cornerstone of safe and effective manual therapy in the management of LBP.

## 2. Materials and Methods

The publication is a literature review based on an analysis of scientific studies on the effectiveness of Kaltenborn-Evjenth, McKenzie methods, and HVLA manipulation techniques in treating lumbosacral spine pain. The review included publications in both Polish and English, available in PubMed, ScienceDirect, PEDro, and the Cochrane Library databases. The search period spanned from 2003 to 2024.

**Figure 1:**
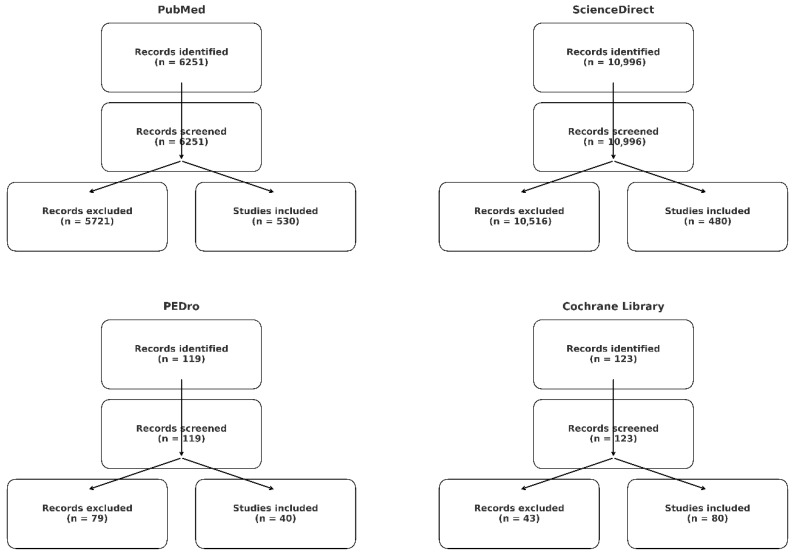
PRISMA 2020 flow diagrams for searches of PubMed, ScienceDirect, PEDro and Cochrane Library.

The primary search was performed using the following key words: (1) “Kaltenborn-Evjenth”[All Fields] AND (“therapeutics”[MeSH Terms] OR “therapeutics”[All Fields] OR “therapies”[All Fields] OR “therapy”[MeSH Subheading] OR “therapy”[All Fields] OR “therapy s”[All Fields] OR “therapys”[All Fields]), (2) (“mckenzie”[All Fields] OR “mckenzie s”[All Fields]) AND (“method s”[All Fields] OR “methods”[MeSH Terms] OR “methods”[All Fields] OR “method”[All Fields] OR “methods”[MeSH Subheading]) AND “low”[All Fields] AND (“back”[MeSH Terms] OR “back”[All Fields]) or (3) “HVLA”[All Fields] AND (“method s”[All Fields] OR “methods”[MeSH Terms] OR “methods”[All Fields] OR “method”[All Fields] OR “methods”[MeSH Subheading]) AND (“low”[All Fields] AND (“back”[MeSH Terms] OR “back”[All Fields])). A PubMed search yielded the following results: 7, 6207, and 37 keywords for searches 1, 2, and 3, respectively. Analogous search of ScienceDirect resulted in 6, 10,605, and 358 for search keywords 1, 2, and 3, respectively. The search of the PEDro database yielded the following results: 3 (clinical trials), 99, and 17 records, respectively. A search of the Cochrane Library yielded 7, 1, and 124 records. The further refinement using the R language, employing specific keywords, resulted in a total of 530, 480, 40, and 80 records, which were then manually evaluated, yielding a final 32 references. The PRISMA flow diagram is shown below in Figure 1.

Final inclusion criteria included studies involving adults with acute, subacute, or chronic lumbar spine pain that evaluated the effectiveness of one of three methods. Short lever manipulation techniques, with clinical indicators such as pain scales (VAS/NPRS), functional performance (ODI, RMDQ), and range of motion (ROM). Randomized clinical trials (RCTs), meta-analyses, and systematic reviews were included. Reports on children and adolescents, as well as case reports and studies without a control group, were excluded from the analysis. Particular attention was paid to the structure of the intervention, its duration, the number of treatment sessions, and the persistence of effects after treatment. The synthesis of the data was descriptive in nature, following the assumptions of the narrative review.

## 3. Results

The Kaltenborn-Evjenth method is more commonly used in the treatment of subacute and chronic lumbar spine pain; however, studies confirm its usefulness in cases of acute pain as well. In a randomized clinical trial, Abdelrahman et al. evaluated the effect of Kaltenborn-Evjenth mobilization on patients with acute lumbar spine pain. The group treated with the Kaltenborn-Evjenth method showed a significantly greater reduction in pain, as measured by the VAS scale, and an improvement in range of motion compared to the control group, which received only posture instruction and general fitness exercises [2].

The use of the Kaltenborn-Evjenth method in chronic lumbar spine pain is a therapeutic challenge due to its complex background, which includes both somatic and psychosocial components. The Kaltenborn-Evjenth method is used in this group of patients primarily due to its precision, low invasiveness, and ability to modify soft tissue tension without causing severe pain reactions. In a study by Shamsi et al., the effects of mobilization using the described method were compared with those of a standard exercise program in 60 patients with chronic lumbar spine pain. The group receiving mobilization achieved significantly greater improvements in flexibility, subjective pain reduction, and function, as measured by the Oswestry Disability Index (ODI) [2]. Another study, conducted by Wegner et al., demonstrated that incorporating Kaltenborn mobilization into a physiotherapy program significantly reduced the time to return to work in patients with chronic low back pain. Importantly, the improvement was also maintained during a 3-month follow-up, which may indicate long-term effects of the therapy. The Kaltenborn-Evjenth method is particularly recommended for people with hypomobility in the lower segments of the spine who experience a lack of flexibility, compensatory tension, and capsular-ligamentous stiffness [3].

The scientific literature contains studies comparing the effectiveness of the Kaltenborn-Evjenth method with other recognized manual therapy techniques, such as the Maitland method, HVLA manipulation techniques, and the McKenzie method. Although the differences are not always clear-cut, the results suggest that Kaltenborn-Evjenth may be equally effective, particularly in improving mobility and reducing segmental stiffness. In a study by Ezzati et al., Kaltenborn and Maitland mobilizations were compared in patients with subacute lumbar spine pain. Both groups reported significant improvement; however, the Kaltenborn-Evjenth group showed faster improvement in range of motion and a greater reduction in muscle tension upon palpation [4]. Fritz et al. compared the Kaltenborn-Evjenth method and HVLA manipulation techniques in the context of mechanical lumbar spine pain. Both methods produced similar results in pain reduction; however, HVLA manipulation techniques worked faster and Kaltenborn-Evjenth more predictably in patients with contraindications to high-velocity manipulation [5]. It is worth noting that the Kaltenborn-Evjenth method often involves a preparatory stage before more intensive techniques, such as HVLA manipulation, are used, or is seen as a safe alternative for older individuals with osteopenia or a fear of manipulation. In combination with stabilization exercises, it can be an effective and individualized form of manual therapy. The findings of the chapter are compiled in Table 1.

Unlike other manual therapy techniques, such as HVLA or McKenzie, the Kaltenborn-Evjenth method has not been as widely analyzed in meta-analyses, partly due to the difficulty of standardizing its techniques and the small number of extensive multicenter studies. Nevertheless, there are systematic reviews that include joint mobilization as part of the treatment for lumbar spine pain. In a review by Akkurt et al., the effectiveness of joint mobilization (including Kaltenborn-Evjenth) in treating chronic lumbar spine pain was evaluated. The conclusions confirmed that these techniques resulted in a significant improvement in function and a reduction in pain compared to conservative treatment alone. Importantly, these effects persisted for at least 6 weeks after the end of therapy [6]. In turn, Stochkendahl et al. reviewed studies on manual interventions and showed that Kaltenborn-Evjenth mobilizations are effective in reducing pain and improving range of motion, especially when combined with patient education and functional exercises [7].

Although the number of meta-analyses focusing exclusively on Kaltenborn-Evjenth techniques is limited, their conclusions are consistent: these mobilizations are effective, safe, and well tolerated by patients. However, there is a need for further studies of high methodological quality using clear treatment protocols. Joint mobilizations in the Kaltenborn-Evjenth method are considered one of the safest manual therapy interventions, especially when compared to HVLA manipulation techniques. These techniques do not require high speed or force, which significantly reduces the risk of damage to anatomical structures [8].

Proper qualification and thorough examination prior to treatment significantly reduce the risk of complications. Correct adherence to procedures and the selection of techniques appropriate to the patient’s condition make the Kaltenborn-Evjenth method a safe therapeutic tool. The work of Studnicki et al. (2024) [9] emphasizes the importance of traction using the Kaltenborn-Evjenth method. The author conducted a randomized study involving 60 people, which clearly demonstrated that manual traction significantly improves function and reduces lumbosacral pain in patients with radicular symptoms, outperforming traditional physiotherapy methods. The results are statistically significant and clinically promising. The authors recommend this approach as an effective treatment for lumbosacral pain with nerve root radiculopathy [9].

Acute lumbar spine pain is characterized by a duration of up to 6 weeks and is most often nonspecific in nature. During this period, nonpharmacological interventions, such as exercise and education, are crucial, especially in reducing the transition of pain to a chronic form [10]. The McKenzie method has gained prominence as an effective, safe, and easy-to-implement treatment for acute lumbar spine pain. One of the most frequently cited studies in this patient group is the work of Long, Donelson, and Fung. The authors conducted a randomized clinical trial involving 312 patients with acute and subacute low back pain. Participants were assigned to one of three groups: treatment in accordance with the preferred direction of the McKenzie method, treatment not in accordance with this direction, and a control group receiving standard recommendations. The results showed that only the group treated in accordance with the preferred direction achieved significant improvement, both in terms of pain reduction and functional improvement [11]. Peterson et al. also showed in a study comparing the McKenzie method with manual therapy that exercises from this method were more effective in reducing pain during the first two weeks of treatment. The effects were particularly pronounced in patients with centralization of symptoms, confirming the high predictive value of this response to movement [12]. In observational studies, Werneke and Hart found that patients with acute lumbar spine pain showed improvement after only 3–5 sessions, and over 70% of them achieved complete pain remission within two weeks, without the need for pain medication or physical therapy [13].

The method is also used in the treatment of chronic lumbar spine pain. Chronic pain is defined as pain that persists for longer than 12 weeks. It is this group of patients that generates the highest treatment costs and most often experiences a deterioration in quality of life. Treatment of chronic lumbar spine pain should be integrated, combining movement, education, and a behavioral approach. The McKenzie method can play a crucial role here because it promotes activity, self-therapy, and patient responsibility in the healing process. In a study by May and Donelson, conducted in an outpatient setting with patients with chronic pain, the use of the McKenzie method led to significant improvement after only 6 weeks. Outstanding results were achieved in patients with centralization of symptoms, even when the pain had persisted for several months. The effects included not only pain reduction but also functional improvement [14,15].

A meta-analysis by Hahne et al. of 14 studies on chronic low back pain also showed that the McKenzie method is at least as effective as stabilization exercises and manual therapy techniques. Moreover, this method was easier to implement, more economical, and associated with greater patient independence in the therapeutic process [16]. It is also worth mentioning the results of a study by Rosedale and Lucente, in which patients with chronic low back pain were classified using the McKenzie method. In individuals treated according to the preferred direction, a significant improvement in the ODI scale and a lower recurrence rate within the next 6 months were observed. The authors emphasized that the effectiveness of the method in this group depends on correct classification and patient involvement in the self-treatment program [16].

Pain radiating to the lower limbs, often referred to as sciatica, is the result of nerve root irritation, usually by a herniated intervertebral disk. Symptoms may include not only pain, but also numbness, muscle weakness, and sensory disturbances. Traditionally, treatment of such cases has focused on pharmacotherapy or surgery. However, the McKenzie method has proven to be an effective, non-invasive alternative. In a study by Albert and Manniche, 174 patients with confirmed intervertebral disk herniation and symptoms of sciatica were evaluated. After 2 weeks of treatment, as many as 67% of participants experienced complete resolution of symptoms and avoided planned surgery. Importantly, most of them experienced centralization, which the authors considered a therapeutic success [17]. In turn, a meta-analysis by May and Aina showed that the McKenzie method leads to faster and more lasting improvement in patients with radiating pain compared to classic exercises and passive therapies. Working with this method not only shortened the duration of neurological symptoms but also reduced the number of pharmacological interventions and the hospitalization rate [18]. Studies by O’Sullivan et al. showed that the use of McKenzie therapy in combination with behavioral education is more effective than manual therapy or pharmacological treatment for pain with a neuropathic component. Patients treated with the McKenzie method were less likely to use pain medication, and their satisfaction with the therapy was significantly higher [19].

To evaluate the effectiveness of the McKenzie method comprehensively, researchers conducted a series of systematic reviews and meta-analyses. They aimed to compare the technique with other methods of treating nonspecific low back pain, using data from multiple randomized clinical trials. One of the most well-known studies is the meta-analysis by Machado et al., which included 13 randomized clinical trials involving over 1600 patients. It was shown that the McKenzie Method is significantly more effective than education and general exercises in reducing pain and improving function in the short term (up to 3 months). Long-term effects (over 6 months) were comparable to those of other forms of treatment, demonstrating the stability of the therapy’s effects [20]. In a meta-analysis by Halliday et al., which included 11 randomized clinical trials, the authors concluded that the McKenzie Method leads to significantly faster functional improvement than passive therapy, physical therapy, or counseling. It was particularly effective in patients with radiating symptoms and a positive centralization response [21].

Clinical studies on the effectiveness of HVLA manipulation in treating acute cases of low back pain have provided promising results. In a randomized controlled trial, Hancock et al. demonstrated that patients with acute lumbosacral pain who received HVLA short-lever manipulation techniques within the first 2 weeks of symptom onset returned to work more quickly and reported greater pain reduction than those treated with conservative therapy [22]. In a study by Childs et al. [23], 131 patients with acute lumbar spine pain were evaluated. In individuals who met the criteria of the so-called “manipulation effectiveness prediction rule” (including a short duration of symptoms and no radiation symptoms), significant pain reduction and functional improvement were achieved after only two sessions of HVLA short-lever manipulation. These results suggested that HVLA manipulation techniques may be particularly effective in the first weeks of symptoms, mainly if patient selection is based on appropriate clinical criteria [23].

Although the effectiveness of this method in treating chronic low back pain was initially controversial, more and more studies confirm its positive effect, even in long-term cases. A survey by Senna and Machaly compared the effectiveness of HVLA manipulation techniques and McKenzie therapy in 148 patients with chronic low back pain. Both groups showed improvement, but the HVLA group had significantly better results in terms of pain reduction after 6 weeks of therapy, which persisted for at least 6 months. However, it should be noted that psychosocial factors (fear of movement, depression) are more critical in patients with chronic pain, which is why they may be decisive in the group treated with HVLA manipulation [24].

Multicenter studies and systematic reviews comparing HVLA manipulation with other physiotherapy techniques suggest that HVLA manipulation techniques are equally effective and, in some cases, more effective than other techniques. In a randomized study, Bronfort et al. compared HVLA manipulation techniques, exercise therapy, and patient education. It was found that spinal manipulation resulted in the fastest reduction in pain and improvement in function, especially in the first weeks of therapy. After one year, the effects of all groups were comparable, suggesting that HVLA manipulation is an effective early intervention method [25]. Walker et al. compared the method in question with segmental mobilization and stabilization exercises. In patients who met the criteria for “manipulation effectiveness,” HVLA produced significantly better results in pain reduction after only 2 weeks. Interestingly, some studies also indicate that HVLA may be as effective as McKenzie therapy in the acute phase of lumbar spine pain; however, the differences become less pronounced in the long term. Therefore, it is crucial to select the proper method for the type of pain, its phase, and the individual characteristics of the patient [26].

Meta-analyses and systematic reviews serve as an essential reference point for assessing the effectiveness of HVLA manipulation, as they synthesize data from multiple studies of varying methodological quality. Several significant works in this area have been published in recent years. Rubinstein et al. conducted a large meta-analysis of 26 randomized controlled trials (RCTs) and concluded that HVLA spinal manipulation techniques are more effective than a placebo and as effective as other active therapies, such as therapeutic exercises or mobilizations. Moreover, manipulations demonstrated a faster analgesic effect, particularly during the first 6 weeks of therapy [27]. In a systematic review, Paige et al. found that lumbar spine manipulation resulted in moderate improvement in pain and function in the lumbar spine, with a low risk of adverse effects. Notably, the effectiveness was comparable to pharmacological therapy, but without its side effects [27]. Gorrell et al. demonstrated that the therapeutic effects of HVLA may be sustained longer when supported by exercise and patient education, confirming the importance of a multimodal approach to treatment. Although these results are promising, the authors of the reviews emphasize the need for further high-quality studies with better standardization of manipulation techniques, patient selection criteria, and longer follow-up periods [6].

The HVLA spinal manipulation technique, performed by trained therapists, is considered a safe method for treating lumbar spine pain. Unlike cervical spine manipulation, which is sometimes associated with more serious complications, lumbar spine manipulation is associated with a low risk of complications. A meta-analysis by Gouveia et al. showed that the incidence of serious adverse events following HVLA manipulation techniques in the lumbar spine is extremely low, approximately 1 in 1,000,000 cases. The most commonly reported symptoms were transient pain, muscle stiffness, fatigue, or dizziness. These symptoms usually resolve within 24–48 h. Comparative studies have shown that HVLA spinal manipulation has a similar or even lower risk of adverse effects than pharmacological treatment with NSAIDs, which can cause severe gastrointestinal and cardiovascular side effects [28].

## 4. Discussion

Comparing the three primary methods of physiotherapy—McKenzie, HVLA, and Kaltenborn-Evjenth—the following conclusions can be drawn. Each method has its own unique therapeutic value and clinical application, and their effectiveness is strongly dependent on the type of patient, pain characteristics, and stage of the disease process.

Kaltenborn-Evjenth mobilization techniques offer the most balanced approach, as they are safe, biomechanically sound, and have proven effectiveness in improving function in patients with limited mobility and chronic pain. Thanks to the gradual dosage of force and the possibility of fine-tuning to the patient’s tolerance level, the method is applicable to a wide range of clinical cases, including those involving the elderly, post-injury, or post-surgery patients. An additional advantage of the Kaltenborn-Evjenth method is that these techniques can be performed both as therapeutic interventions and diagnostic tests [29,30].

The McKenzie method demonstrates the highest effectiveness in patients with mechanical types of pain and centralization phenomena, as well as in those who are willing to participate in therapy actively. Importantly, it aligns with modern models of chronic pain treatment, which emphasize patient autonomy and the reduction in passive forms of therapy. The method works well not only as a therapy for symptoms, but also allows the patient to gain a better understanding of the nature of their problems and prevent recurrence. For this reason, it is widely used in the treatment of both acute and chronic lumbosacral spine pain [31,32].

HVLA manipulation techniques, although somewhat controversial in light of recent research, remain one of the fastest methods for achieving improved function and reduced pain, especially for individuals experiencing acute episodes with restricted motion. Numerous studies have confirmed that HVLA therapy causes immediate improvements in mobility and stimulates the neurophysiological response of the nervous system, thereby reducing spinal muscle activity. The effectiveness of this method primarily depends on the therapist’s precision and experience, as well as the patient’s careful assessment. Therefore, it is very often used as a complementary element rather than the main form of therapy. An absolute prerequisite for safety in this case is the observance of contraindications to the procedure (e.g., tumors, infections, fractures, advanced osteoporosis). Therefore, short-lever HVLA manipulation should only be performed by individuals with appropriate clinical training and experience [32,33]. The compilation of the findings of this section is gathered in Table 1.

## 5. Conclusions

The most effective approach is not one based solely on a single method, but rather one that integrates the advantages of several methods. In an acute episode, HVLA manipulation techniques can be used, followed by McKenzie exercises. In chronic lumbosacral spine pain, it is advisable to start with mobilization using the Kaltenborn-Evjenth method, and then incorporate educational elements and stand-alone exercises. A common element of effective treatment is to involve the patient in their decision-making process, educate and motivate them to participate actively in therapy. Each method can be effective, but its effect depends on the clinical context, not just the technique itself. Findings from numerous studies and systematic reviews suggest that a multimedia therapeutic approach, combining manual techniques, exercise, and education, yields the best results for patients with lumbar spine pain.

## Figures and Tables

**Table 1:** Comparative Summary of Kaltenborn-Evjenth, McKenzie, and HVLA Techniques in the Treatment of Lumbar Spine Pain.

Parameter	Kaltenborn-Evjenth Mobilization	McKenzie Method (MDT)	HVLA Manipulation
Indications	Chronic/subacute LBP, segmental hypomobility, soft tissue tension	Acute and chronic LBP, directional preference, centralization signs	Acute LBP, mobility restriction, short symptom duration
Mechanism of Action	Passive joint mobilization, soft tissue elongation, neurophysiological inhibition	Repeated movements, self-correction, centralization	Rapid joint release, mechanical and reflex effects
Pain Relief	Moderate to strong, gradual onset	Strong, especially in patients with centralization	Strong, typically immediate in acute cases
Functional Improvement	Yes, especially in chronic cases with stiffness	Yes, in both acute and chronic cases	Yes, rapid in acute cases with appropriate selection
Typical Duration of Therapy	6–12 sessions	3–10 sessions; emphasizes self-management	1–5 sessions, depending on response
Contraindications	Severe instability, malignancy, infection	Rare; limited mainly to non-mechanical pain types	Osteoporosis, fractures, nerve root compression, malignancy
Evidence Quality	Moderate; mostly RCTs and narrative reviews	High; supported by multiple RCTs and meta-analyses	High in acute LBP; mixed for chronic LBP
Advantages	Safe, precise, well tolerated, useful for chronic conditions	Patient empowerment, active therapy, cost-effective	Fast pain relief, useful for acute phase
Limitations	Slower onset of effects, requires clinical skill	Less effective in non-mechanical or complex chronic pain	Requires training, greater risk if misapplied
Long-Term Effectiveness	Maintained with follow-up exercises	Well supported, especially with self-management	Effective if combined with education/exercise

## Data Availability

Not applicable.
